# Cancer risk in persons with new-onset anaemia: a population-based cohort study in Denmark

**DOI:** 10.1186/s12885-022-09912-7

**Published:** 2022-07-21

**Authors:** Astrid Boennelykke, Henry Jensen, Lene Sofie Granfeldt Østgård, Alina Zalounina Falborg, Anette Tarp Hansen, Kaj Sparle Christensen, Peter Vedsted

**Affiliations:** 1 Research Unit for General Practice, Bartholins Allé 2, DK-8000 Aarhus C, Denmark; 2 Department of Public Health, Aarhus University, Bartholins Allé 2, DK-8000 Aarhus C, Denmark; 3 Department of Haematology, Odense University Hospital, J.B. Winsloews Vej 4, DK-5000 Odense C, Denmark; 4 Department of Clinical Epidemiology, Aarhus University Hospital, Palle Juul-Jensens Boulevard 99, DK-8200 Aarhus N, Denmark; 5 Department of Clinical Biochemistry, Aalborg University Hospital, Hobrovej 18, DK-9100 Aalborg, Denmark

**Keywords:** Anemia, Cancer risk, Cohort studies, Denmark, General practice

## Abstract

**Background:**

The time interval from first symptom and sign until a cancer diagnosis significantly affects the prognosis. Therefore, recognising and acting on signs of cancer, such as anaemia, is essential. Evidence is sparse on the overall risk of cancer and the risk of specific cancer types in persons with new-onset anaemia detected in an unselected general practice population. We aimed to assess the risk of cancer in persons with new-onset anaemia detected in general practice, both overall and for selected cancer types.

**Methods:**

This observational population-based cohort study used individually linked electronic data from laboratory information systems and nationwide healthcare registries in Denmark. We included persons aged 40–90 years without a prior history of cancer and with new-onset anaemia (no anaemia during the previous 15 months) detected in general practice in 2014–2018. We measured the incidence proportion and standardised incidence ratios of a new cancer diagnosis (all cancers except for non-melanoma skin cancers) during 12 months follow-up.

**Results:**

A total of 48,925 persons (median [interquartile interval] age, 69 [55–78] years; 55.5% men) were included in the study. In total, 7.9% (95% confidence interval (CI): 7.6 to 8.2) of men and 5.2% (CI: 4.9 to 5.5) of women were diagnosed with cancer during 12 months. Across selected anaemia types, the highest cancer incidence proportion was seen in women with ‘anaemia of inflammation’ (15.3%, CI: 13.1 to 17.5) (ferritin > 100 ng/mL and increased C-reactive protein (CRP)) and in men with ‘combined inflammatory iron deficiency anaemia’ (19.3%, CI: 14.5 to 24.1) (ferritin < 100 ng/mL and increased CRP). For these two anaemia types, the cancer incidence across cancer types was 10- to 30-fold higher compared to the general population.

**Conclusions:**

Persons with new-onset anaemia detected in general practice have a high cancer risk; and markedly high for ‘combined inflammatory iron deficiency anaemia’ and ‘anaemia of inflammation’. Anaemia is a sign of cancer that calls for increased awareness and action. There is a need for research on how to improve the initial pathway for new-onset anaemia in general practice.

**Supplementary Information:**

The online version contains supplementary material available at 10.1186/s12885-022-09912-7.

## Introduction

Cancer is a leading cause of death in several countries, and detecting cancer at an early stage is associated with improved survival [1, 2]. Early detection requires recognition of signs and symptoms to facilitate timely investigation and lower mortality [3, 4]. Around three in four persons diagnosed with cancer initially present in general practice, and these persons are often associated with low cancer risk as they often present with non-specific signs [4, 5]. Even persons with recognized alarm symptoms of cancer may indicate a low cancer risk (e.g. 1.4% in persons with unexpected weight loss) [6–8]. This challenges the clinical interpretation.

Anaemia is a non-specific sign of possible cancer, and anaemia occurs in 17% of persons aged 65+ years [9]. Iron deficiency anaemia is a well-established marker of increased risk of gastrointestinal cancer (1–10%), [10–13] and necessitates investigation in certain age groups [14]. Further, anaemia occurs in 39% of persons diagnosed with cancer with domination of mild anaemia (75%) [15]. Moreover, anaemia is a negative prognostic factor for survival of several cancer types [16].

Anaemia may be present in a variety of underlying diseases. Nevertheless, the evidence is sparse on the cancer risk in persons with new-onset anaemia in an unselected general practice population. Although anaemia of inflammation (AI) and iron deficiency anaemia (IDA) are the most common anaemia types, [17] poor evidence exists on the overall cancer risk in these persons [18, 19]. AI is associated with comorbidities, and often referred to as chronic anaemia. Yet, the evidence is weak on the cancer risk in new-onset AI in general practice. A single study revealed that 23% of persons with AI in general practice had cancer [19]. However, this study was limited by excluding persons with an unestablished cause and various length of follow-up within a 6-year period. Another study investigated the cancer risk in persons with IDA in general practice and revealed a nearly six-fold increased cancer incidence compared to the general population [18]. However, this study was limited by defining IDA through register codes. Moreover, concomitant inflammation may be present in persons with IDA, and inflammatory markers are associated with an increased cancer risk [20]. Still, the cancer risk in this category of anaemia is unknown.

In a large population-based cohort study, we aimed to establish the overall risk of cancer and the risk of specific cancer types in persons with new-onset anaemia detected in general practice.

## Methods

### Design and data sources

**Fig. 1:**
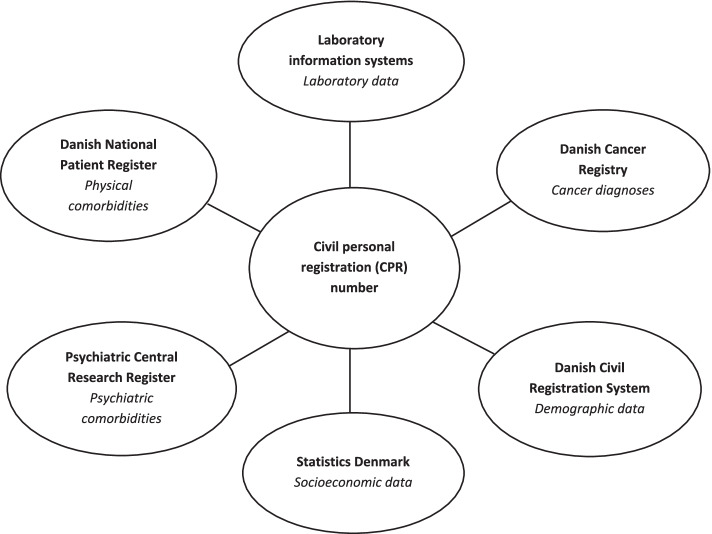
Sources of data linked at the individual level

We performed an observational population-based cohort study using electronic data from Danish laboratory information systems [21] and nationwide healthcare registries [22–24]. The unique civil personal registration (CPR) number (assigned to all Danish residents) allows accurate individual-level linkage of these data (Fig. 1). The laboratory systems hold information on all blood test results requested in general practice or at hospitals and analysed at a department of clinical biochemistry. The Cancer Registry holds information on all cancers diagnosed in Denmark, including cancer type and time of diagnosis. The National Patient Register and the Psychiatric Central Research Register hold information on all hospital contacts and diagnoses in Denmark. The Civil Registration System and Statistics Denmark hold demographic and socioeconomic data on all residents in Denmark.

### Setting

This study is based on data from persons living in the North Denmark Region (0.6 million inhabitants) or the Central Denmark Region (1.3 million inhabitants), which are two of the five Danish regions. We used the laboratory systems in these two regions, as these comprise complete laboratory data within the study period (in contrast to the nationwide laboratory database) [21, 25]. Danish residents (5.8 million inhabitants) listed with a specific general practice (99%) have unrestricted access to healthcare free of charge, and the general practitioner (GP) serves as gatekeepers and coordinators to the specialised healthcare (except for emergencies, ear-nose-throat specialists, and eye specialists) [22, 26].

### Identification of the study cohort

The inclusion criteria were: (i) 40–90 years of age at the date of anaemia (as we considered this age group to be clinically relevant for considerations of cancer), (ii) new-onset anaemia detected by a blood test requested from general practice and recorded in the laboratory systems, (iii) date of new-onset anaemia between 1 April 2014 and 31 December 2017, and (iv) living in one of the two included regions at inclusion.

We did not allow persons to re-enter the cohort, and we excluded persons with a prior history of cancer recorded in the Cancer Registry.

### Variables

#### Exposure

The exposure was new-onset anaemia detected in general practice. We defined anaemia as a haemoglobin level below 134 g/L for men and below 118 g/L for women in accordance with the Danish reference intervals [27]. We defined new-onset anaemia as no anaemia registered in the laboratory systems, regardless of origin of request, in the up to 15 months preceding the anaemia date in the inclusion period (to exclude patients with chronic anaemia having annual consultations). We defined the date of new-onset anaemia in the inclusion period as the index date.

Based on blood tests obtained in general practice within 31 days of the index date, we categorized new-onset anaemia into anaemia types based on the guideline for unexplained anaemia by the Danish Society for Gastroenterology and Hepatology [28, 29]. They comprised four etiological anaemia types: (i) AI (ferritin > 100 microgram/L (μg/L) and increased C-reactive protein (CRP)), (ii) combined inflammatory iron deficiency anaemia (CIIDA) (ferritin < 100 μg/L and increased CRP), (iii) IDA (ferritin < 30 μg/L regardless of the CRP level), and (iv) ‘other’ (i.e. other anaemia aetiological causes) (ferritin > 30 μg/L and normal CRP) [28, 29]. If the anaemia could not be categorised into one of these four groups due to lacking blood tests, the anaemia was categorised under a fifth category: ‘unclassified’.

#### Outcomes

The main outcome measure was a cancer diagnosis within a 12-month follow-up period together with a graphical presentation of the monthly increase in cancer diagnosis. All cancers (classified according to the International Classification of Diseases, 10th revision (ICD-10)) were included, except for non-melanoma skin cancers (ICD-10 code C44). The different cancer types were divided into eight specific cancer groups: breast cancer, cancer in the kidney and urinary tract system, cancer in the respiratory system, gastrointestinal cancer, gynaecological cancer, haematological cancer, male genital cancer, and other cancers. Only the first cancer diagnosis for each individual was included in the analyses within each of the specific cancer groups and for overall cancer, respectively.

#### Characteristics of study population

The study population was characterized by sex, age, educational level, disposable income, civil status, anaemia severity, and comorbidity burden. Age was categorized as 40–49 years, 50–59 years, 60–69 years, 70–79 years, and 80–89 years. Adjustments for age were performed using restricted cubic splines with three knots [30]. Educational level was categorized as ‘low’, ‘medium’, and ‘high’ according to the International Standard Classification of Education (ISCED) [31]. Disposable income was categorized into tertiles of ‘low’, ‘medium’, and ‘high’. Civil status was categorized into ‘living with a partner’ and ‘living alone’. Anaemia severity was categorized into ‘mild’, ‘medium’, and ‘severe’ according to the definitions by the World Health Organization [32]. Comorbidity registered in hospitals within 10 years preceding the index date was included. Number of comorbidities (equally weighted) was categorized into none, one, two, and three or more. Comorbidity was categorized into ten chronic disease groups, including arthritis, cardiovascular disease, chronic obstructive pulmonary disease, diabetes, hypertension, inflammatory bowel disease, kidney disease, liver disease, mental illness, and neurological disorders,. These disease groups have been used in previous research, [33–37] and the specific comorbidities included in the different groups are displayed in supplemental material in a previous paper [36].

### Statistical analysis

We estimated the time to a cancer diagnosis from the date of new-onset anaemia. We assessed the incidence proportion of cancer based on the Aalen-Johansen estimator, considering death as a competing risk. We used the Aalen-Johansen estimator instead of the Kaplan Meier estimator as we considered death as a competing event, and Kaplan-Meyer estimates are biased on data with competing risks [38]. We followed all persons until a cancer diagnosis, death, emigration from the included regions, or end of 12- month follow-up, whichever came first. We stratified analyses by anaemia type and sex.

Further, to compare the cancer incidence in the study population to a general population, we estimated standardised incidence ratios (SIRs) of cancer and of specific cancer types based on age- and sex-specific cancer incidence rates in a general population by use of the NORDCAN database [39, 40]. NORDCAN is a database of cancer statistics for the Nordic countries, and includes information on e.g. cancer incidence [39, 40]. We included a general Danish population aged 40 to 85+ years, and used estimated 12-month cancer incidence rates in a comparable period from 2014 to 2018 [39, 40]. We stratified the analyses by anaemia type and sex.

To identify persons at increased risk of cancer, we estimated the associations between patient characteristics and risk of cancer. We assessed the hazard ratios (HRs) by applying a multivariable Cox proportional hazard model, treating competing risk (death) as censoring. We evaluated the proportional hazard assumption from log-minus log plots, and we detected no violation of the assumptions. We followed the persons until any first cancer diagnosis, emigration from the included regions, death, or end of 12- month follow-up, whichever came first. We stratified the analyses by anaemia types and adjusted for age (continuous), anaemia severity, civil status, educational level, income, comorbidity, and sex. Missing values occurred in the variables with educational level (*n =* 1979, 4.0%) and income (*n =* 203, 0.4%), and were handled as representing ‘low educational level’ and ‘low income’. All persons (*n =* 48,925*)* were registered in the Civil Registration System, and thereby no loss to follow-up.

This study followed the STROBE reporting guideline. We performed all analyses in Stata**®** version 16.

## Results

**Fig. 2:**
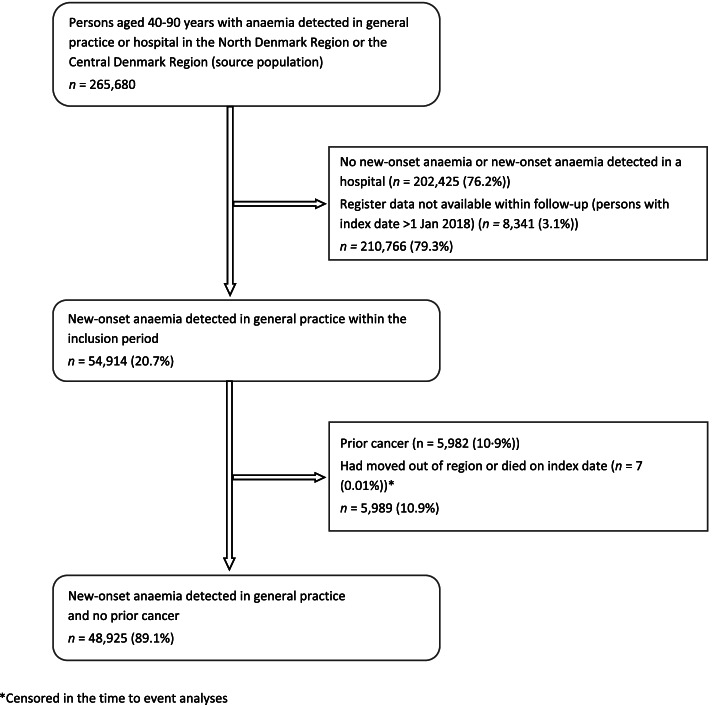
Flowchart of the study population

We included 48,925 persons with new-onset anaemia in the analyses (Fig. 2). The median age was 69 years (interquartile interval (IQI) 55–78) (men: 70 years, IQI 60–78, women: 66 years, IQI 48–79), and 55.5% (27,148) were men. Overall, 78.3% (38,286) had mild anaemia, and 46.0% (22,522) had comorbidities; the most common being cardiovascular disease (24.9%, 12,173) and hypertension (21.7%, 10,604) (Table 1). In total, the number of person years was 45,105, and the number of cancer cases was 3285 (6.7%).

**Table 1:** Demographic and clinical characteristics of persons with new-onset anaemia according to anaemia types

Patient characteristics	AI n (%)	CIIDA n (%)	IDA n (%)	Other n (%)	Unclassified n (%)	Total n (%)
**Total, n (%)**^**a**^	2640 (5.4)	639 (1.3)	7674 (15.7)	4570 (9.3)	33,402 (68.3)	48,925 (100.0)
**Age groups, years**						
40–49	165 (6.3)	83 (13.0)	3386 (44.1)	555 (12.1)	4211 (12.6)	8400 (17.2)
50–59	362 (13.7)	68 (10.6)	1355 (17.7)	825 (18.1)	4773 (14.3)	7383 (15.1)
60–69	657 (24.9)	142 (22.2)	981 (12.8)	1037 (22.7)	7054 (21.1)	9871 (20.2)
70–79	835 (31.6)	180 (28.2)	1127 (14.7)	1185 (25.9)	9424 (28.2)	12,751 (26.1)
80–89	621 (23.5)	166 (26.0)	825 (10.8)	968 (21.2)	7940 (23.8)	10,520 (21.5)
**Anaemia severity** ^**b**^						
Mild	1984 (75.2)	445 (69.6)	3244 (42.3)	3871 (84.7)	28,742 (86.0)	38,286 (78.3)
Moderate	620 (23.5)	181 (28.3)	3883 (50.6)	658 (14.4)	4302 (12.9)	9644 (19.7)
Severe	36 (1.4)	13 (2.0)	547 (7.1)	41 (0.9)	358 (1.1)	995 (2.0)
**Civil status**						
Living with a partner	1454 (55.1)	315 (49.3)	4372 (57.0)	2609 (57.1)	18,730 (56.1)	27,480 (56.2)
Living alone	1186 (44.9)	324 (50.7)	3302 (43.0)	1961 (42.9)	14,672 (43.9)	21,445 (43.8)
**Educational level**						
Low	1203 (45.6)	333 (52.1)	2905 (37.9)	1893 (41.4)	14,798 (44.3)	21,132 (43.2)
Medium	1003 (38.0)	215 (33.6)	2888 (37.6)	1787 (39.1)	12,773 (38.2)	18,666 (38.2)
High	434 (16.4)	91 (14.2)	1881 (24.5)	890 (19.5)	5831 (17.5)	9127 (18.7)
**Income**						
Low	996 (37.7)	248 (38.8)	1988 (25.9)	1535 (33.6)	11,484 (34.4)	16,251 (33.2)
Medium	859 (32.5)	217 (34.0)	2342 (30.5)	1424 (31.2)	11,274 (33.8)	16,116 (32.9)
High	785 (29.7)	174 (27.2)	3344 (43.6)	1611 (35.3)	10,644 (31.9)	16,558 (33.8)
**No. of comorbidities**						
0	1516 (57.4)	309 (48.4)	5185 (67.6)	2484 (54.4)	16,909 (50.6)	26,403 (54.0)
1	592 (22.4)	150 (23.5)	1379 (18.0)	1111 (24.3)	8238 (24.7)	11,470 (23.4)
2	350 (13.3)	105 (16.4)	696 (9.1)	650 (14.2)	5246 (15.7)	7047 (14.4)
≥ 3	182 (6.9)	75 (11.7)	414 (5.4)	325 (7.1)	3009 (9.0)	4005 (8.2)
**Sex**						
Men	1612 (61.1)	259 (40.5)	1448 (18.9)	2885 (63.1)	20,944 (62.7)	27,148 (55.5)
Women	1028 (38.9)	380 (59.5)	6226 (81.1)	1685 (36.9)	12,458 (37.3)	21,777 (44.5)
**Type of comorbidity** ^**c**^						
Arthritis	38 (1.4)	6 (0.9)	52 (0.7)	47 (1.0)	343 (1.0)	486 (1.0)
Cardiovascular disease	606 (23.0)	164 (25.7)	1177 (15.3)	1126 (24.6)	9100 (27.2)	12,173 (24.9)
COPD	170 (6.4)	75 (11.7)	302 (3.9)	224 (4.9)	2072 (6.2)	2843 (5.8)
Diabetes	190 (7.2)	73 (11.4)	575 (7.5)	397 (8.7)	3697 (11.1)	4932 (10.1)
Hypertension	522 (19.8)	152 (23.8)	1086 (14.2)	979 (21.4)	7865 (23.5)	10,604 (21.7)
IBD	17 (0.6)	12 (1.9)	72 (0.9)	47 (1.0)	334 (1.0)	482 (1.0)
Liver disease	46 (1.7)	20 (3.1)	78 (1.0)	64 (1.4)	417 (1.2)	625 (1.3)
Mental illness	190 (7.2)	69 (10.8)	606 (7.9)	368 (8.1)	3060 (9.2)	4293 (8.8)
Neurological disorder	68 (2.6)	20 (3.1)	136 (1.8)	150 (3.3)	1082 (3.2)	1456 (3.0)
Kidney disease	44 (1.7)	12 (1.9)	50 (0.7)	68 (1.5)	592 (1.8)	766 (1.6)

*Abbreviations*: *AI* Anaemia of inflammation, *CIIDA* Combined inflammatory iron deficiency anaemia, *COPD* Chronic obstructive pulmonary disease, *IBD* Inflammatory bowel disease, *IDA* Iron deficiency anaemia, *No* Number, *Unclassified* The anaemia is not classifiable according to a guideline

^a^Total percentages are shown in row percentages, other variables are shown in column percentages

^b^Anaemia severity was defined according to WHO’s guidelines: mild anaemia (haemoglobin > 110 g/L), moderate anaemia (haemoglobin 80–110 g/L), and severe anaemia (haemoglobin < 80 g/L)

^c^Comorbidity was registered for the ten years preceding the index date and categorized according to the chronic disease groups

### Incidence proportion

A total of 7.9% (CI = 7.6–8.2) of men and 5.2% (CI = 4.9–5.5) of women were diagnosed with cancer within 12 months. Gastrointestinal cancer was the most frequent cancer type in both men (2.7%, CI = 2.5–2.9) and women (2.2%, CI = 2.1–2.4).

**Fig. 3:**
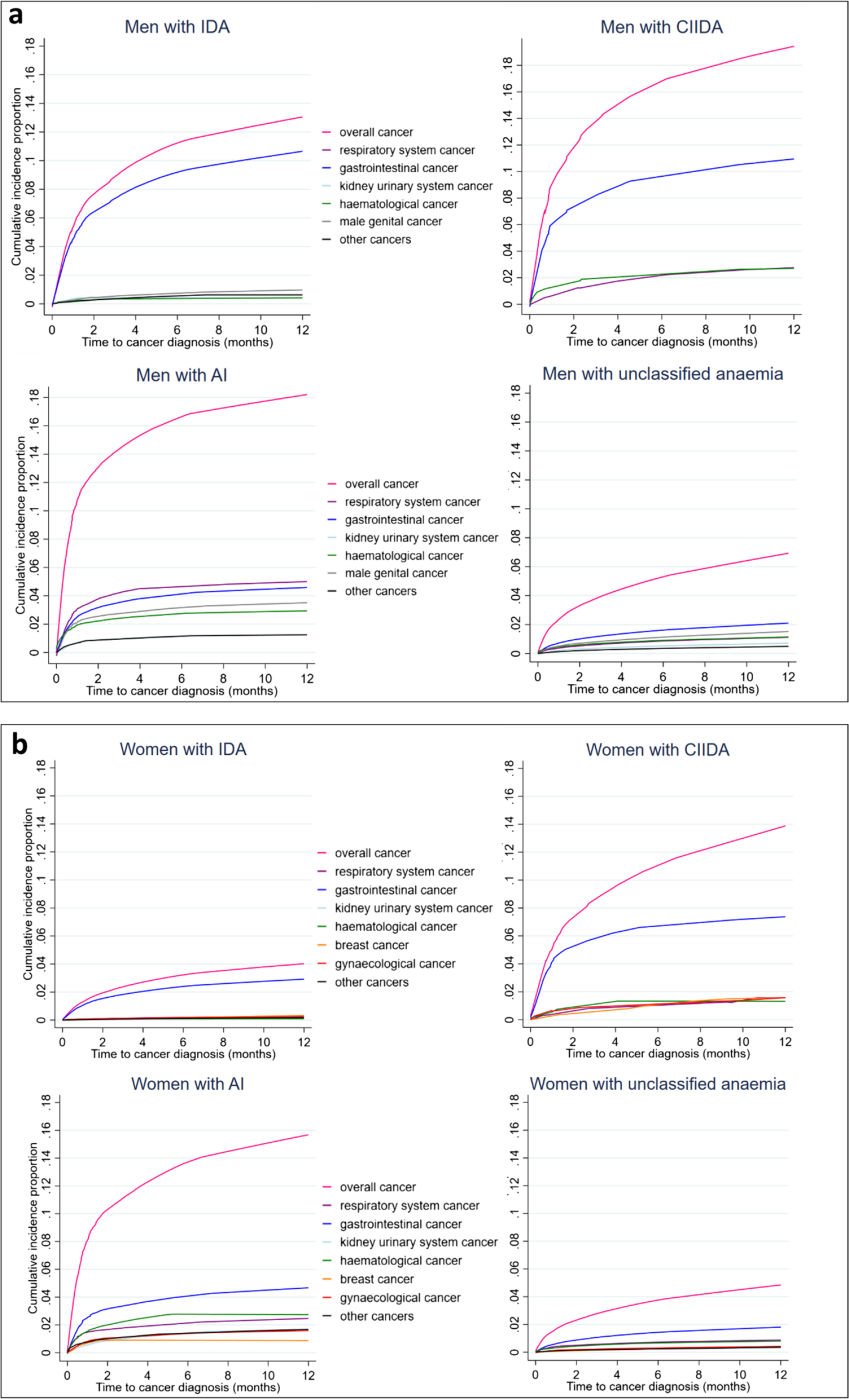
a Cumulative cancer incidence in men with new-onset anaemia during 12-month (by anaemia types). Abbreviations: AI: anaemia of inflammation, CIIDA: combined inflammatory iron deficiency anaemia, IDA: iron deficiency anaemia, Unclassified anaemia: the anaemia is not classifiable according to a guideline. For overall cancer risk: all cancers were included, except for non-melanoma skin cancer. Proportions with < 5 events are not shown to protect confidentiality.3b. Cumulative cancer incidence in women with new-onset anaemia during 12-month (by anaemia types). Abbreviations: AI: anaemia of inflammation, CIIDA: combined inflammatory iron deficiency anaemia, IDA: iron deficiency anaemia, Unclassified anaemia: the anaemia is not classifiable according to a guideline. For overall cancer risk: all cancers were included, except for non-melanoma skin cancer. Proportions with < 5 events are not shown to protect confidentiality.

Across the anaemia types, the highest cancer incidence proportion was seen in men with CIIDA (19.3%, CI = 14.5–24.1) and women with AI (15.3%, CI = 13.1–17.5) (Fig. 3a, Fig. 3b). Gastrointestinal cancer was the most frequent cancer type in persons with IDA, CIIDA, AI (women) and unclassified anaemia; the highest proportion was seen in men with CIIDA (10.8%, CI = 7.0–14.6). Respiratory system cancer was the most frequent cancer type in men with AI (5.0%, CI = 3.9–6.0), whereas haematological cancer was the most frequent cancer type in persons with ‘other’ anaemia (men 1.8%, CI = 1.3–2.3, women 1.9%, CI = 1.2–2.6).

Most cancers were diagnosed 3–6 months after the new-onset anaemia (Fig. 3a, Fig. 3b).

### Standardised incidence ratios

**Fig. 4:**
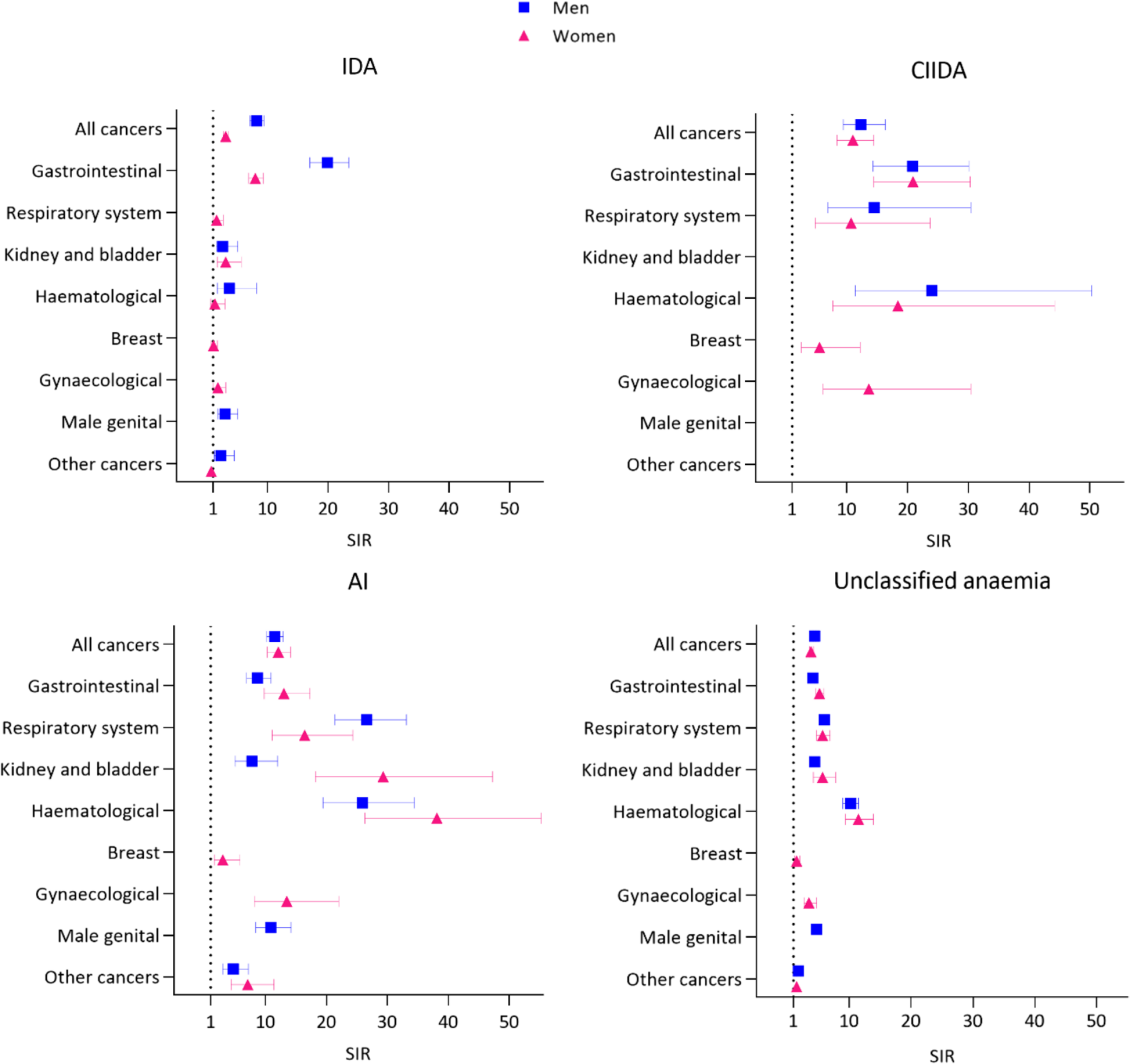
Standardized incidence ratios^a^ for cancer^b^ in persons with new-onset anaemia (by anaemia types). Abbreviations: AI: anaemia of inflammation, CI: 95% confidence interval, CIIDA: combined inflammatory iron deficiency anaemia, IDA: iron deficiency anaemia, SIR: Standardized incidence ratios, Unclassified: the anaemia is not classifiable according to a guideline. SIRs with < 5 events are not shown to protect confidentiality. Error bars = 95% CI (some error bars are not seen because of a narrow 95% CI). ^a^Based on age- and sex-specific cancer incidence rates in the general Danish population (from the NORDCAN database). ^b^Non-melanoma skin cancer excluded

The SIRs for overall cancer was 5.1 (CI = 4.9–5.3) in men and 4.1 (CI = 3.9–4.4) in women (Fig. 4). The cancer type with the highest SIR was haematological cancer in both men (SIR 11.5, CI = 10.4–12.8) and women (SIR 11.1, CI = 9.6–12.9).

Across the anaemia types, the highest SIR for overall cancer was seen in men with CIIDA (SIR 12.3, CI = 9.4–16.3) and in women with AI (SIR 12.1, CI = 10.3–14.1). Across the cancer types, the highest SIR was seen for respiratory system cancer in men with AI (SIR 26.6, CI = 21.4–31.1) and haematological cancer in women with AI (SIR 38.1, CI = 26.3–55.2) (Fig. 4).

### Associations between patient characteristics and cancer

**Fig. 5:**
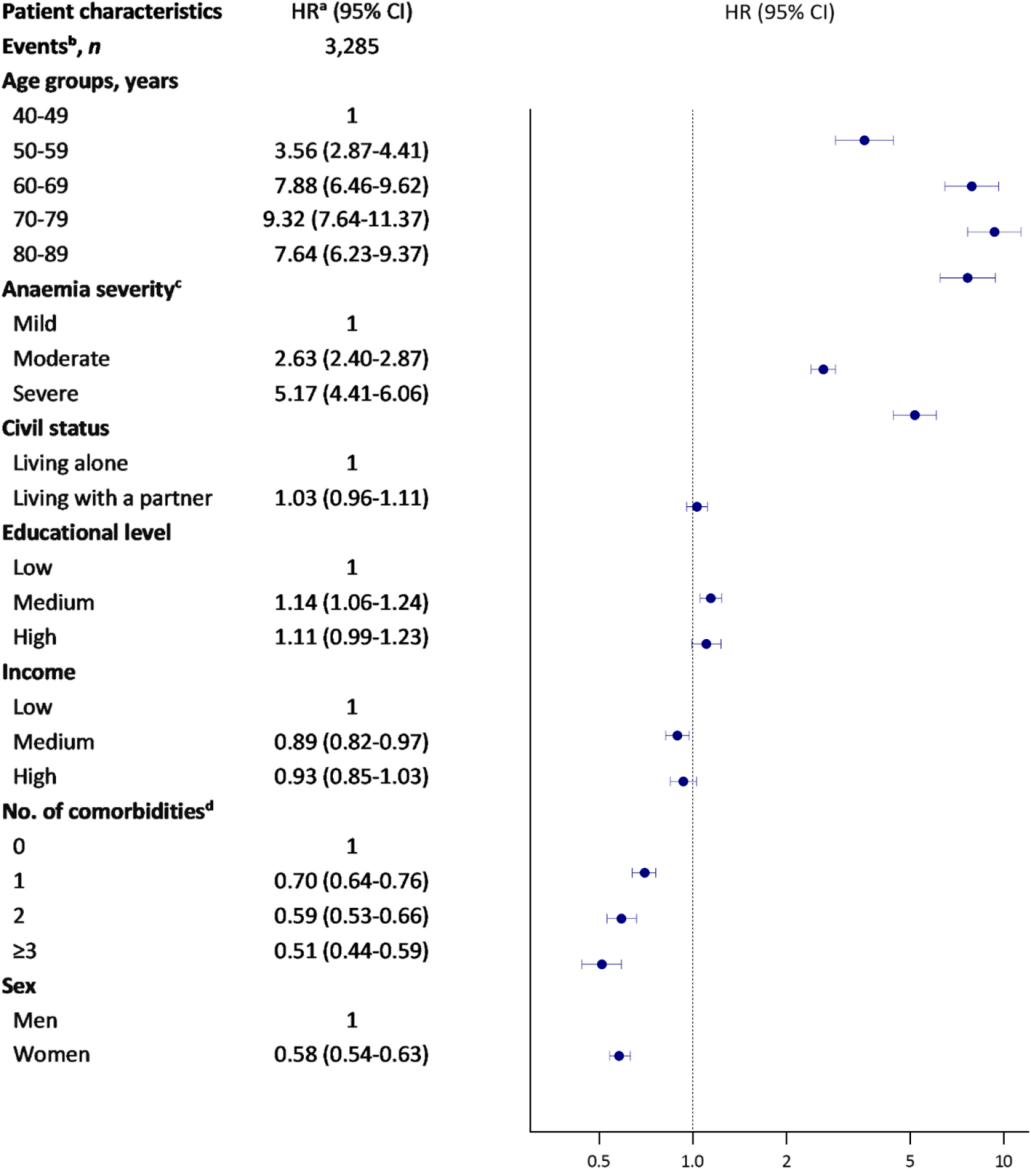
Associations of patient characteristics and cancer in persons with new-onset anaemia. Abbreviations: CI: 95% confidence interval, HR: hazard ratio, No: number. Error bars = 95% CI (some error bars are not seen because of a narrow 95% CI). Hazard ratios are displayed on a log scale. ^a^Adjusted for age (continuous), anaemia severity, civil status, educational level, income, comorbidity, and sex. ^b^Non-melanoma skin cancer excluded. ^c^Anaemia severity was defined according to WHO’s guidelines: mild anaemia (haemoglobin > 110 g/L), moderate anaemia (haemoglobin 80–110 g/L), and severe anaemia (haemoglobin < 80 g/L). ^d^Comorbidity was registered ten years prior to the index date and categorized according to the chronic disease groups (CDGs)

Persons aged 70–79 years were more likely to get cancer compared to persons aged 40–49 years (HR 9.32, CI = 7.64–11.37) (Fig. 5); the highest likelihood was seen in persons aged 70–79 years with IDA (HR 18.32, CI = 11.96–28.09) (Table 2). Persons with severe anaemia were more likely to get cancer compared to persons with mild anaemia (HR 5.17, CI = 4.41–6.06); the highest likelihood was seen in persons with unclassified severe anaemia (HR 6.47, CI = 5.10–8.21). Women were less likely to get cancer compared to men (HR 0.58, CI = 0.54–0.63); the lowest likelihood was seen in women with IDA (HR 0.44, CI = 0.36–0.54). Persons with three or more comorbidities were less likely to get cancer compared to persons without comorbidity (HR 0.58, CI = 0.54–0.63) (Fig. 5); the lowest likelihood was seen in persons with CIIDA and three or more comorbidities (HR 0.25, CI = 0.10–0.64) (Table 2).

**Table 2:** Associations of patient characteristics and cancer in persons with new-onset anaemia (by anaemia types)

Patient characteristics	AI HR^a^ (95% CI)	CIIDA HR^a^ (95% CI)	IDA HR^a^ (95% CI)	Other HR^a^ (95% CI)	Unclassified HR^a^ (95% CI)
**Events,** ***n*** **(%)**^b**,c**^ **Person years**	446 (13.6%) 2118	103 (3.1%) 528	429 (13.1%) 7239	255 (7.8%) 4295	2052 (62.5%) 30,923
**Age groups, years**					
40–49	1	1	1	1	1
50–59	1.58 (0.90–2.76)	2.50 (0.78–7.98)	3.34 (2.06–5.39)	3.37 (1.49–7.63)	3.10 (2.31–4.17)
60–69	2.14 (1.27–3.62)	3.68 (1.35–10.06)	11.77 (7.64–18.14)	6.39 (2.92–13.98)	6.51 (4.95–8.56)
70–79	2.46 (1.46–4.16)	9.50 (3.48–25.98)	18.32 (11.96–28.09)	6.95 (3.14–15.34)	7.24 (5.51–9.51)
80–89	1.86 (1.08–3.23)	3.70 (1.25–10.94)	16.81 (10.74–26.30)	7.41 (3.31–16.59)	6.05 (4.59–8.00)
**Anaemia severity** ^**d**^					
Mild	1	1	1	1	1
Moderate	1.87 (1.49–2.35)	2.00 (1.31–3.06)	1.87 (1.49–2.34)	2.43 (1.75–3.37)	2.87 (2.54–3.25)
Severe	3.66 (2.09–6.40)	1.22 (0.29–5.11)	4.05 (3.07–5.36)	2.72 (1.01–7.35)	6.47 (5.10–8.21)
**Civil status**					
Living alone	1	1	1	1	1
Living with a partner	1.10 (0.89–1.35)	0.98 (0.66–1.46)	1.13 (0.92–1.39)	1.17 (0.88–1.55)	1.03 (0.94–1.13)
**Educational level**					
Low	1	1	1	1	1
Medium	1.18 (0.96–1.46)	1.80 (1.15–2.81)	1.08 (0.86–1.35)	1.50 (1.12–2.01)	1.11 (1.00–1.23)
High	1.04 (0.77–1.40)	1.36 (0.69–2.67)	1.40 (1.05–1.86)	1.23 (0.84–1.81)	1.08 (0.94–1.24)
**Income**					
Low	1	1	1	1	1
Medium	1.08 (0.85–1.36)	0.62 (0.38–1.04)	0.82 (0.64–1.04)	0.95 (0.68–1.31)	0.89 (0.80–0.99)
High	0.99 (0.76–1.29)	0.95 (0.53–1.30)	0.88 (0.67–1.16)	1.17 (0.84–1.64)	0.91 (0.80–1.03)
**No. of comorbidities** ^**e**^					
0	1	1	1	1	1
1	0.48 (0.37–0.63)	0.48 (0.28–0.81)	0.70 (0.55–0.89)	0.64 (0.46–0.88)	0.77 (0.69–0.86)
2	0.62 (0.46–0.85)	0.58 (0.33–1.03)	0.52 (0.38–0.72)	0.71 (0.49–1.03)	0.60 (0.52–0.69)
≥ 3	0.44 (0.28–0.71)	0.25 (0.10–0.64)	0.59 (0.40–0.87)	0.48 (0.26–0.86)	0.54 (0.45–0.65)
**Sex**					
Men	1	1	1	1	1
Women	0.68 (0.54–0.84)	0.69 (0.45–1.04)	0.44 (0.36–0.54)	0.61 (0.45–0.83)	0.60 (0.53–0.67)

*Abbreviations*: *AI* Anaemia of inflammation, *CI* 95% confidence interval, *CIIDA* Combined inflammatory iron deficiency anaemia, *HR* Hazard ratio, *IDA* Iron deficiency anaemia, *No* Number, *Unclassified* The anaemia is not classifiable according to a guideline

^a^Adjusted for age (continuous), anaemia severity, civil status, educational level, income, comorbidity, and sex

^b^Non-melanoma skin cancer excluded

^c^Row percentages

^d^Anaemia severity was defined according to WHO’s guidelines: mild anaemia (haemoglobin > 110 g/L), moderate anaemia (haemoglobin 80–110 g/L) and severe anaemia (haemoglobin < 80 g/L)

^e^Comorbidity was registered ten years prior to the index date and categorized according to the chronic disease groups (CDGs)

As a supplement to the HRs, number of cancer cases stratified by patient characteristics and anaemia types are shown in an Additional file (see Additional Table 1).

## Discussion

### Principal findings

This population-based cohort study of nearly 49,000 persons with new-onset anaemia detected in general practice revealed a risk of cancer in 7.9% of men and 5.2% of women within 12 months. Around one in six persons with ‘anaemia of inflammation’ or ‘combined inflammatory iron deficiency anaemia’ got a cancer diagnosis within 12 months. About one in ten of the cancers occurred in the group with IDA. Thus, the majority of cancers came from outside this group traditionally investigated for gastrointestinal cancer. The cancer incidence increased particularly in the first 2–3 months after the anaemia date followed by a significantly slower increase for some cancer types, which could indicate diagnostic activity. For some of the anaemia types, the cancer incidence continued to increase during all 12 months after the anaemia date.

Among the new-onset anaemias, a four- to five-fold higher cancer incidence occurred in women and men compared to the general population. Among the anaemia types, an 11- to 12-fold higher overall cancer incidence occurred in men and women with ‘anaemia of inflammation’ or ‘combined inflammatory iron deficiency anaemia’ compared to the general population. Across cancer types, these two anaemia types had a 10- to 30-fold higher cancer incidence compared to the general population.

### Strengths and limitations

This large-scale population-based cohort study holds individually linked data from nationwide registries and laboratory systems known for a high validity and completeness [21, 24]. This ensured virtually complete follow-up with limited risk of selection bias and information bias [21, 24]. Further, the general practice setting, including an unselected population with free access to healthcare services, [22] makes the results widely relevant and may be generalized to other countries with similar access to the healthcare system and with comparable populations (it may not be generalized to other socioeconomic or geographical settings with a higher prevalence of anaemia related diseases, e.g. Thalassaemia in Middle East countries or malnutrition in Africa).

We lacked information on potential and unavailable confounding factors, e.g. smoking and obesity. Thus, we cannot rule out the potential of residual confounding. Further, in the multivariable Cox regression analysis, there is a potential risk of overfitting of the analysis in the smallest group with CIIDA. Furthermore, we had no information on the reasons for the persons to consult their GP, and we do not know what prompted the GP to investigate for an anaemia. Thus, this may introduce confounding by indication as persons having encounters with their GP and having blood tests performed are likely to be more ill compared to the general population. However, haemoglobin measurement is one of the most frequently performed blood tests [41]. This may indicate that non-specific or opportunistic screening for anaemia may be the indication in many cases. If so, this may indicate that this confounding factor may be less dominant. The Cox model treated competing risk (death of any cause) as censoring. However, this was likely informative censoring which could bias the results. Moreover, detection bias might have occurred because clinicians may be aware of anaemia as a sign of cancer, and this paradox might have led to diagnostic evaluation and cancer detection. However, previous research has revealed that the laboratory and diagnostic process of new-onset anaemia in general practice is suboptimal [36, 37]. Therefore, this may reduce the potential risk of detection bias. All these factors imply that we regard the findings as exploratory rather than causal.

The unselected population and the significantly increased cancer risk after new-onset anaemia make it important to establish the aetiological reason for the anaemia. Still, the majority of new-onset anaemia cases were unclassified, [36] and it is unknown which anaemia type these may represent. The proportion of unclassified anaemias in other countries is unknown and has not previously been included when exploring the cancer risk in selected anaemia types. Additional research is needed to explore this large group of persons having unclassified anaemia in general practice.

### Comparison with other studies

To our knowledge, this is the first large-scale study to investigate the overall risk of cancer and the risk of specific cancer types across selected anaemia types detected in a general practice population with new-onset anaemia, including CIIDA and unclassified anaemia.

Previous studies have mainly focused on anaemia/IDA and gastrointestinal cancer [10–13]. These have reported a risk of gastrointestinal cancer of 1–10%, which is in accordance with our findings [10–13]. Additionally, the one-year SIR for overall cancer in persons with IDA has previously been reported to be 6.12 (CI = 5.57–6.78) in men and 5.60 (CI = 5.13–6.11) in women [18]. Compared to our findings, these figures are slightly lower in men and slightly higher in women. This could be due to different standard populations and other definitions of IDA. Another study reported a cancer incidence of 9.8% (CI = 8.6 to 11.1) in men and 4.0% (CI = 3.3–4.9) in women with microcytic anaemia (common in persons with IDA), [42] which is comparable to our findings.

Previous research has rarely focused on the risk of overall cancer and specific cancer types in persons with AI in a general practice population [19]. A single and small-scale study showed that 23% of persons with AI had underlying malignancy, [19] which is higher than our findings. However, this may be due to different lengths of follow-up.

Previous findings on cancer risk and characteristics in persons with anaemia/IDA in general practice are in line with our findings [10, 11, 13]. Furthermore, the highest cancer risk occurred in CIIDA and AI, which are both characterized by an underlying inflammation. Inflammatory markers are associated with an increased cancer risk, and the risk increases as the level of inflammation rises [20]. Thus, the combination of inflammation and anaemia should increase the clinical alertness of underlying cancer. Moreover, across all investigated anaemia types, we found that persons with comorbidities were less likely to be diagnosed with cancer compared to persons without comorbidity. A reasonable explanation could be that patients with comorbidities may already have a reasonable explanation for the anaemia (i.e. comorbidity associated with anaemia, such as kidney disease and rheumatologic disease) [17, 43]. However, research seems needed on the cancer risk in persons with anaemia having certain comorbidities associated with an increased cancer risk, such as diabetes, [44–46] cardiovascular disease, [45, 47] and inflammatory bowel disease [48].

Further, we found that women had a lower cancer risk compared to men. This could reflect that anaemia is a benign sign in premenopausal women due to e.g. menstrual bleeding. However, in a sub-analysis on the cancer risk in persons aged 50–90 years, the cancer risk in women compared to men increased only marginally (HR 0.86 vs. HR 0.84). This may reflect the overall increased cancer incidence in men compared to women, which is well-established and has been seen in the past decades in the Nordic countries [40].

## Conclusions and implications

Diagnosing cancer at an early stage is a high priority to clinicians, patients, and public. Therefore, recognising signs of possible cancer is essential. Our findings indicate that new-onset anaemia is an important sign of possible cancer in an unselected general practice population; this sign requires high awareness among health professionals, especially when seen in persons with AI and CIIDA, who had a strikingly high risk of cancer.

Nonetheless, previous research found that the majority of persons with new-onset anaemia had insufficient blood tests performed to allow categorisation of the anaemia into IDA, CIIDA, and AI [36]. Furthermore, despite clinical recommendations of referral of persons with unexplained anaemia to an urgent cancer patient pathway in Denmark, evidence indicate that this is not yet clinical practice [37]. Thus, improved clinical practice (e.g. by trigger algorithms and cancer-risk assessment tools) for persons with anaemia is needed and may have important prognostic implications [49]. This calls for interventional research including assessment of the cancer stage and prognosis of persons with new-onset anaemia diagnosed with cancer. Moreover, future research seems needed on the cancer risk in different age groups and the long-term cancer risk in persons with new-onset anaemia.

## Supplementary Information

**Additional file 1: Table 1. ** Number of cancer cases in persons with new-onset anaemia by patient characteristics and anaemia types.

## Data Availability

The data used in this study is stored and anonymised on the servers of Statistics Denmark. As restrictions apply to the availability of these data, we used it under license for the current study. Therefore, the data is not publicly available, but it may be available upon formal request (please contact the corresponding author).

## Abbreviations

AI: Anaemia of inflammation
CI: Confidence interval
CIIDA: Combined inflammatory iron deficiency anaemia
CPR number: Civil personal registration number
CRP: C-reactive protein
GP: General practitioner
HR: Hazard ratio
ICD-10: International Classification of Diseases, 10th revision
IDA: Iron deficiency anaemia
IQI: Interquartile interval
SIR: Standardized incidence ratio
