# Supplementary material for: Cancer risk in persons with new-onset anaemia: a population-based cohort study in Denmark
Source: BMC Cancer. 2022 Jul 21;22:805. doi: 10.1186/s12885-022-09912-7 (PMC9306185; doi:10.1186/s12885-022-09912-7)
Supplement: Supplementary file 1 — Additional file 1: Table 1. Number of cancer cases in persons with new-onset anaemia by patient characteristics and anaemia types. [file 12885_2022_9912_MOESM1_ESM.docx]

**Additional table 1.** *Number of cancer cases in persons with new-onset anaemia by patient characteristics and anaemia types*

| **Patient characteristics** | **AI** | **CIIDA** | **IDA** | **Other** | **Unclassified** |
| --- | --- | --- | --- | --- | --- |
| **Total, *n* (%)^a,b^** | 446 (13.6%) | 103 (3.1%) | 429 (13.1%) | 255 (7.8%) | 2,052 (62.5%) |
| **Age groups, years** |  |  |  |  |  |
| 40-49 | 16 (3.6%) | 5 (4.9%) | 29 (6.7%) | 7 (2.8%) | 59 (2.9%) |
| 50-59 | 52 (11.7%) | 7 (6.8%) | 40 (9.3%) | 33 (12.9%) | 187 (9.1%) |
| 60-69 | 126 (28.3%) | 21 (20.4%) | 98 (22.8%) | 74 (29.0%) | 541 (26.4%) |
| 70-79 | 167 (37.4%) | 53 (51.5%) | 164 (38.2%) | 81 (31.8%) | 764 (37.2%) |
| 80-89 | 85 (19.1%) | 17 (16.5%) | 98 (22.8%) | 60 (23.5%) | 501 (24.4%) |
| **Anaemia severity^c^** |  |  |  |  |  |
| Mild | 300 (67.3%) | 61 (59.2%) | 136 (31.7%) | 194 (76.1%) | 1,579 (77.0%) |
| Moderate | 133 (29.8%) | 40 (38.8%) | 212 (49.4%) | 57 (22.4%) | 400 (19.5%) |
| Severe | 13 (2.9%) | - | 81 (18.9%) | - | 73 (3.6%) |
| **Civil status** |  |  |  |  |  |
| Living alone | 182 (40.8%) | 50 (48.5%) | 182 (42.4%) | 89 (34.9%) | 840 (40.9%) |
| Living with a partner | 264 (59.2%) | 53 (51.5%) | 247 (57.6%) | 166 (65.1%) | 1,212 (59.1%) |
| **Educational level** |  |  |  |  |  |
| Low | 185 (41.5%) | 47 (45.6%) | 196 (45.7%) | 86 (33.7%) | 883 (43.0%) |
| Medium | 188 (42.2%) | 42 (40.8%) | 147 (34.3%) | 119 (46.7%) | 824 (40.2%) |
| High | 73 (16.4%) | 14 (13.6%) | 86 (20.1%) | 50 (19.6%) | 345 (16.8%) |
| **Income** |  |  |  |  |  |
| Low | 174 (39.0%) | 47 (45.6%) | 178 (41.5%) | 96 (37.7%) | 826 (40.3%) |
| Medium | 142 (31.8%) | 29 (28.2%) | 134 (31.2%) | 69 (27.1%) | 656 (32.0%) |
| High | 130 (29.2%) | 27 (26.2%) | 117 (27.3%) | 90 (35.3%) | 570 (27.8%) |
| **No. of comorbidities^d^** |  |  |  |  |  |
| 0 | 315 (70.6%) | 65 (63.1%) | 265 (61.8%) | 158 (62.0%) | 1,160 (56.5%) |
| 1 | 64 (14.4%) | 18 (17.5%) | 86 (20.1%) | 50 (19.6%) | 489 (23.8%) |
| 2 | 48 (10.8%) | 15 (14.6%) | 48 (11.2%) | 35 (13.7%) | 266 (13.0%) |
| ≥3 | 19 (4.3%) | 5 (4.9%) | 30 (7.0%) | 12 (4.7%) | 137 (6.7%) |
| **Sex** |  |  |  |  |  |
| Men | 289 (64.8%) | 50 (48.5%) | 185 (43.1%) | 182 (71.4%) | 1,445 (70.4%) |
| Women | 157 (35.2%) | 53 (51.5%) | 244 (56.9%) | 73 (28.6%) | 607 (29.6%) |

Abbreviations: AI: anaemia of inflammation, CIIDA: combined inflammatory iron deficiency anaemia, IDA: iron deficiency anaemia, No.: number, Unclassified: the anaemia is not classifiable according to a guideline.

^a^Non-melanoma skin cancer excluded.

^b^Total percentages are shown in row percentages, other variables are shown in column percentages.

^c^Anaemia severity was defined according to WHO’s guidelines: mild anaemia (haemoglobin >110 g/L), moderate anaemia (haemoglobin 80-110 g/L) and severe anaemia (haemoglobin <80 g/L).

^d^Comorbidity was registered ten years prior to the index date and categorized according to the chronic disease groups (CDGs).

Numbers with <5 events are not shown.
